# Coexistence of Rheumatoid Arthritis, Systemic Lupus Erythematosus, Sjogren Syndrome, Antiphospholipid Syndrome, and Ankylosing Spondylitis

**DOI:** 10.1155/2021/8491717

**Published:** 2021-08-10

**Authors:** Moshiur Rahman Khasru, Md Abu Bakar Siddiq, Kazi Mohammad Sayeeduzzaman, Tangila Marzen, Abul Khair Mohammad Salek

**Affiliations:** ^1^Department of Physical Medicine and Rehabilitation, Bangabandhu Sheikh Mujib Medical University, Dhaka-1000, Bangladesh; ^2^Department of Physical Medicine and Rehabilitation, Brahmanbaria Medical College, Brahmanbaria, Bangladesh; ^3^Department of Dental Radiology, Shaheed Suhrawardy Medical College, Dhaka-1207, Bangladesh

## Abstract

A 37-year-old Bangladeshi woman presented with low back and several joints pain and swelling for months together; there was significant morning stiffness for more than two hours. Repeated abortions, dry eye, hair fall, photosensitivity, and oral ulcer were the additional complaints. Clinical examination unveiled asymmetrical peripheral and both sacroiliac joint tenderness, positive modified Schober's test, and limited chest expansion. Schirmer's test was positive. The history of rheumatoid arthritis (RA) and ankylosing spondylitis (AS) among 1st-degree relatives was also significant. Biochemical analysis revealed pancytopenia, raised erythrocyte sedimentation rate (ESR) and C-reactive protein (CRP), and mild microscopic proteinuria. The patient was seropositive for rheumatoid factor (RF), antibodies against cyclic citrullinated peptides (anti-CCP), antinuclear antibody (ANA), anti-Sm antibody, anti-Sjögren's-syndrome-related antigen A and B (anti-SSA/SSB), antiphospholipid (aPL-IgG/IgM), and HLA B27; however, serum complement (C3 and C4) levels were normal. Basal cortisol level measured elevated. Besides, X-ray and MRI of lumbosacral spines demonstrated sacroiliitis. There was radiological cardiomegaly, echocardiography unveiled atrial regurgitation, and ascending aorta aneurysm. Based on the abovementioned information, RA, AS, and systemic lupus erythematosus (SLE) have been diagnosed. Moreover, the patient developed Sjogren's syndrome (SS), antiphospholipid lipid syndrome (APS), Cushing syndrome, ascending aorta aneurysm, and atrial regurgitation. Her disease activity score for RA (DAS28), DAS for AS (ASDAS), SLE disease activity index (SLEDAI), and Systemic Lupus International Collaborating Clinics/American College of Rheumatology (SLICC/ACR) scores were 3.46, 2.36, 23, and 5, respectively. The patient received hydroxychloroquine (200 mg daily), pulsed cyclophosphamide, prednisolone (20 mg in the morning), and naproxen 500 mg (twice daily). To our best knowledge, this is the first report documenting RA, AS, and SLE with secondary SS and APS.

## 1. Introduction

Ankylosing spondylitis (AS) and rheumatoid arthritis (RA) are the two most common autoimmune chronic inflammatory rheumatic diseases that result in progressive joint destruction and disabilities [1]. Ankylosing spondylitis (AS) is a member of the prototypical autoimmune disease group known as spondyloarthropathies (SpAs) [2]. Several classification criteria have been developed, of which ASAS criteria (Assessment of SpondyloArthritis International Society) and modified New York criteria are widely used in the diagnosis of AS [3, 4]. The etiopathogenesis of both AS and RA is not well established yet; however, the genetic associations of these disorders are different. HLA B27 is associated with AS, whereas HLA DR4 is associated with RA [5]. However, a significant role in disease pathogenesis is played by immunological factors. The typical clinical features of AS may include inflammatory back pain, asymmetrical oligoarthritis, enthesitis, anterior uveitis, a significant reduction in Schober's test, radiological sacroiliitis, and positive for HLA B27 [6].

The annual incidence of AS ranges from 5 to 140 per million per year, with a prevalence between 1 and 14 per thousand [6]. AS has a twice higher prevalence among men compared to women [7]. RA classically featured with symmetrical polyarthritis involving small joints of the hands and feet. Early classification of RA can be made using the 2010 American College of Rheumatology (ACR)/European League against Rheumatism (EULAR) classification criteria of RA. The presence of antibodies against cyclic citrullinated peptides (anti-CCP) in the serum is definite for RA [8].

On the other hand, systemic lupus erythematosus (SLE) is a various austere form of the autoimmune rheumatic disease that may present with remarkably varied clinical manifestations [9]. However, diagnosis can be made by using ‘2019 EULAR/ACR classification criteria for SLE [10]. Several autoantibodies such as antinuclear antibodies (ANA), anti-Sjögren's-syndrome-related antigen A and B (anti-SSA/SSB), antibodies directed against double-stranded DNA (anti-dsDNA), anti-Smith (anti-Sm), small nuclear ribonucleoprotein (snRNP), and antiphospholipid antibody (aPL) are associated with SLE [11]. Furthermore, Sjogren's syndrome (SS) may be associated with SLE and RA [12]. Lacrimal and salivary glands are involved in SS, resulting in dry eye and mouth symptoms as primary clinical manifestations [13, 14]. Antiphospholipid syndrome (APS) is characterized by recurrent vascular thrombosis and loss of pregnancy, and the presence of aPL: APS can be primary and secondary to other systemic disease, for example, SLE [15]. Sporadic case reports described overlap syndrome of SLE-AS, and SLE-RA (rhupus). However, to be our best knowledge, we are yet to have a complicated case scenario demonstrating the simultaneous presence of RA, AS, SLE, SS, and APS; in the present study, we perform it for the first time in the medical literature in a 37-year-old woman. Informed written consent has been obtained from the family (husband of the patient with an eye witness of her elder brother) for publication of a case report.

## 2. Case Report

A 37-year-old Bangladeshi woman with Cushingoid face presented with complaints of inflammatory low back and several large and small joint pain and swelling that affected symmetrically the upper extremities involving both the wrists and bilateral 2nd and 3rd metacarpophalangeal (MCP) joint and asymmetrically in the lower limbs involving the right knee and both ankle joints with significant morning stiffness longer than one hour for the last four years. Pain aggravated during inactivity and rest and partially relieved with activity and steroidal and nonsteroidal anti-inflammatory drugs (NSAIDs) such as indomethacin and naproxen. Moreover, she complained of headaches, shortness of breath (exertional and lying bed), malaise, and anorexia; however, the patient had no leg swelling. There was a history of repeated spontaneous miscarriage at the first trimester of pregnancy, itching, and foreign-body sensation in the eyes and alopecia, though the history of photosensitivity, rash, reddening of the eye, oral ulcer, tingling and numbness of both the upper and lower limbs, and loss of weight or contact with a TB patient was insignificant. She had a positive family history of RA and AS in her 1st-degree relatives. AS and RA was diagnosed: AS in an elder brother and nephew and RA in a younger sister. Painless mouth ulcer involved the tongue, soft palate, and gum.

She was anemic (hemoglobin 8.7 gram/dl, range 13.5–16.00), and she had a puffy face. She neither had cough, palpitation, vomiting of blood, or chest pain. Her bowel and bladder habits were also normal. Her vital signs were stable, though her breathing pattern was reversed (abdominothoracic). Besides, grade II tenderness elicited over sacroiliac joints. Modified Schober's test and chest expansion and occiput-wall distance were recorded as 4, 2, and 1 cm, respectively. However, the Owen Thomas test was negative. She neither had a rounded shoulder, protruded abdomen, flat chest, or stooping forward posture while standing or walking, typical AS phenotype. Examination of higher psychic function and cranial nerves unveiled no abnormality.

Biochemical investigations unveiled low hemoglobin, microcytic hypochromic anemia, low hematocrit (30%), thrombocytopenia, leucopenia, and raised ESR (40 mm in 1st hour, range 0–10), raised CRP (24 mg/dl, reference value >3), and mild proteinuria were seen. Schirmer's test was positive. She was positive for RA (test value 41.4 U/ml, reference value <16), anti-CCP (test value 42.5 U/ml, reference value-<5 U/ml), ANA (performed in indirect immunofluorescence on Hep-2 cell and titer was 1 : 320), anti-Sm antibody, anti-SSA, anti-SSB, antiphospholipid IgG/IgM (anticardiolipin and anti-b2glycoprotein1), and HLA B27, but prothrombin time (PT), partial thromboplastin time (PTT), and dilute Russell viper venom test (DRVVT) were normal. She was negative for anti-dsDNA, anti- RNP, anti-Jo-1 (antibody is directed against the histidyl-tRNA synthetase), anti- Scl70 (antitopoisomerase antibody type of ANA), antineutrophil cytoplasmic antibodies (p-ANCA and c-ANCA), and Coomb's test (used to detect antibodies that act against the surface of red blood cells). Screening for quantiferon TB gold, ICT for malaria, and ICT for filarial was uneventful. Her serum thyroid status and complements (C3 and C4) levels were within normal values; however, she had elevated serum basal cortisol level (1203 nmol/L (reference range 140–690 nmol/L), and IgE was 175.4 IU/ml (cut off value <100)).

**Figure 1:**
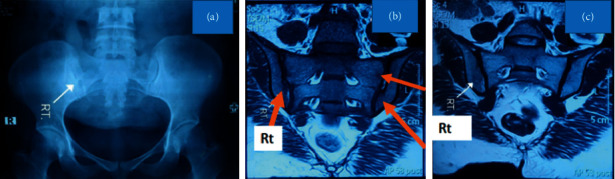
Sacroiliitis. (a) X-ray pelvis shows right-sided sacroiliitis. (b, c) Bilateral sacroiliitis on MRI. Photo: courtesy of the patient.

**Figure 2:**
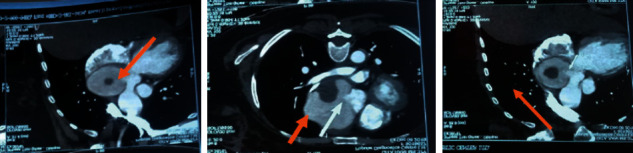
CT angiogram of the heart and great vessels. Large eccentric structure is noted in the root of the ascending aorta having fistulous communication. A hypodense area is noted within the structure. The structure is compressing the corresponding superior vena cava and left ventricular outlet or aortic root (7.5 mm), suggestive of large aneurysm at the aortic root containing the thrombus. Photo: courtesy of the patient.

Radioimaging included X-ray sacroiliac joint that revealed grade II bilateral sacroiliitis (grade III right side, grade II left side) (Figure 1(a)). MRI also confirmed bilateral sacroiliitis (Figures 1(b) and 1(c)). CXR showed cardiomegaly; echocardiography unveiled atrial regurgitation and ascending aorta aneurysm, with an ejection fraction of 62%. CT angiogram of the heart and great vessels further explored a large aneurysm with thrombus at the aortic root (Figure 2). High-resolution computed tomography (HRCT) depicted scattered fibrosis at the inferior segment of the left upper lobe favoring interstitial lung disease (ILD).

RA (ACR 2010 score was 8), AS (according to modified New York criteria), SLE (according to ACR and SLICC criteria), SS (according to 2002 American-European criteria, score 4/6, including serology for anti-SSA/SSB), and APS (based on clinical and immunology profile) were the ultimate diagnosis. Her disease activity score (DAS28), ASDAS, SLE disease activity index (SLEDAI), and Systemic Lupus International Collaborating Clinics/American College of Rheumatology (SLICC/ACR) scores was 3.46, 2.36, 23, and 5, respectively. A multidisciplinary approach was made to treat the patient. The patient is under hydroxychloroquine 200 mg daily, prednisolone 20 mg in the morning, and naproxen 500 mg twice daily with omeprazole and calcium supplementation. Pulsed cyclophosphamide (CYC) was given. Unfortunately, we lost the patient due to a massive hemorrhagic stroke from ruptured aneurysm after two weeks of the 1st dose of CYC; at that time, she developed thrombocytopenia (130 thousand/mm^3^ (reference value 150–450)).

## 3. Discussion

Overlap syndromes (OSs) satisfy clinical and biochemical features of at least two connective tissue diseases (CTDs) sequentially or simultaneously [16]. CTDs include SLE, RA, idiopathic inflammatory myositis (dermatomyositis, DM, polymyositis, and PM), systemic sclerosis (SSc), and Sjogren's syndrome (SS). Literature unveils the existence of every combination between these CTDs [16, 17]. An OS is not a mere association of two or more CTDs in the same patient, rather a well-defined clinical entity with specific clinical characteristics; in OS, clinical features may be different from those observed in the single CTD [16]. OSs provide unique opportunities to understand links between autoimmunity and end-organ immune targeting [17]. Cirstea et al. described a case of a 39-year-old Canadian woman's known case of OS (SLE, SS, APS, and DM) reported to the Dermatology Clinic of Emergency County Hospital for ulcerative lesions on the left hallux and the second and third left toes because of digital vasculopathy with impaired ambulation that improved with intravenous synthetic analogue of prostacyclin PGI2-iloprost and local therapeutic measures [18]. In a cohort among Colombians, APS is found to coexist with other ADs, mostly with SLE, 9.3% of the total study population. The association is linked with a higher cardiovascular (CVD) and pulmonary involvement suggested to require early preventive and therapeutic intervention [19].

Rhupus is an overlap between SLE and RA [20–22]. Devrimsel and Serdaroglu Beyazal described three patients who shared clinical and serological characteristics of rhupus [20]. All satisfied the ACR criteria for SLE and RA. Besides symmetrical polyarthritis, they were positive for ANA, anti-CCP, anti-ds-DNA (2/3 patients), and anti-SSA, and anti-Sm (one patient each). SLE usually develops as nonerosive arthritis; however, rhupus arthritis could be erosive as seen in the present study [20]. In another study, Benavente presented the clinical and serological characteristics of four rhupus patients [22]. They fulfilled ACR criteria for SLE and RA: ANA positive with titers ranging from 1/80 to 1/5250 and anti-ds-DNA, anti-CCP (3/4), and positive antiphospholipid antibodies in two patients (anticardiolipin and lupus anticoagulant) without manifestation APS. All had symmetrical erosive polyarthritis. One patient presented renal affection and 2 with subcutaneous nodules. Two patients were refractory to conventional DMARD and required biologics and mycophennolate mofetil [22]. Fernández et al. reported eight patients with rhupus arthropathy; among them, three had erosive arthritis. All were positive for RA and ANA. They argued that rhupus is not a combination of RA and SLE, rather should be regarded as a variant of lupus arthropathy [21].

Combination of different CTDs in the same patient has been reported in the literature; however, a combination of more than one CTDs with seronegative arthropathy, for example, AS, is not well reported. Tarhan et al. [1] and Akbaryan and Soltani [9] reported SLE-AS in a Turkish and an Iranian woman, respectively. In another case study, such AS in association with discoid lupus has also been reported in a Turkish male [23]. Barczyńska et al. described the RA-AS combination in a case series of three Polish people [24]. In another study, Xu et al. demonstrated an association between SAPHO (Synovitis-Acne-Pustulosis-Hyperstosis-Osteitis) syndrome and RA in a 59-year-old woman, and the duration between the two diagnoses was 10 years [25].

In the present study, we report RA, SLE, and AS based on clinical and laboratory findings simultaneously in the same patient; the patient also developed APS and SS and could be linked with RA and SLE pathology. Besides, the patient had Cushing syndrome, ascending aorta aneurysm, and atrial regurgitation.

Secondary APS is well reported in SLE; however, it could also happen with RA as well as demonstrated by O'Leary et al. in a recent case study. In the study, in contrast to high-aPL-antibody titers and thrombotic events as seen in SLE, the association of high aPL and cutaneous ulceration with necrosis was apparent in a 39-year-old woman, a severe RA patient [26]. APS in association with SLE may have frequent arthralgias, arthritis, autoimmune hemolytic anemia, livedo reticularis, epilepsy, glomerular thrombosis, and myocardial infarction and should address them during management. Considering vascular events, the prognosis of SLE patients with APS/aPL is still worse than that of SLE patients with negative aPL and, hence, require special attention [15].

In the present study, previous miscarriage, aortic root thrombus, and high-aPL titers could signify APS; however, the patient had no skin manifestations. In CTD, shortness of breath (SOB) signifies either involvement of the lungs, developing pulmonary artery hypertension and or anemia; in the present case, HRCT-depicted ILD and anemia could link with her SOB.

SS could also be developed secondarily in SLE and RA [27, 28]. In a recent SLE cohort, one-quarter of cases had SS (SLE-SS) and the frequency was found to be increased with age. A majority were positive for SSA/SSB; however, 39% were seronegative for SSA/SSB. SLE-SS shared many features with primary SS, including female predominance, late disease onset, and neuropathy [27]. The occurrence of SS in RA may have worse outcomes with increased morbidity and mortality as revealed among 83 Iranians in a recent cross-sectional study: the prevalence of SS was 5.9%, and positive unstimulated whole salivary flow rate (USWSFR) and the Lissamine test were more common in RA patients complicated with secondary SS compared to those without SS (*p*=0.01); sicca symptoms were associated with UWSFR (*p*=0.01) [28].

According to Kurata et al., aortic aneurysm is a rare cardiac complication of lupus [24, 29]. Diagnosis and treatment of cardiopulmonary involvement in CTD may be delayed due to its ambiguity of presenting symptoms and is associated with increased morbidity and mortality and poor survival [30]. Therefore, careful attention should be paid to identify patients with CTD to identify features of cardiopulmonary involvement. Increased IgE production has been documented in various autoimmune diseases, including SLE; high levels of autoreactive IgE could relate with increased SLE activity and lupus nephritis and may serve as a potential predictor of SLE [31]. Longitudinal study could assess whether any link between increased IgE and SLE onset, flare up, and lupus nephritis, and the same could be true for our study case as well. Ruptured cerebral aneurysm in SLE is rare; however, rapid aneurysm could develop and rupture in an SLE, as demonstrated by Graffeo et al., and could be true for our study subject as well. So, careful long-term follow-up of aneurysms and treating them aggressively with endovascular embolization and/or open surgical clipping should be considered when it appears appropriate [32].

Cojocaru et al. [33] demonstrated multiple autoimmune syndromes (three or more conditions) are of three types: type 1 (myasthenia gravis, thymoma, PM, and giant cell myocarditis), type 2 (SS, RA, primary biliary cirrhosis, scleroderma, and autoimmune thyroid disease), and type 3 (autoimmune thyroid disease, myasthenia gravis and/or thymoma, SS, pernicious anemia, idiopathic thrombocytopenic purpura, Addison's disease, type 1 diabetes mellitus, vitiligo, autoimmune hemolytic anemia, SLE, and dermatitis herpetiformis) [33]. Our case does not fall into any one category; hence, revision of the autoimmune syndromes classification could be carried out.

In conclusion, the simultaneous presence of several CTDs in the same patient has been reported in the medical literature; however, combining more than one CTD in association with seronegative arthritis in the same patient has not been well studied and requir further exploration. We also mentioned rapid growth of aneurysm and its rupture could terminate patient survival.

## Data Availability

Data can be obtained from the corresponding author.

## Additional Points

The coexistence of several connective tissue diseases such as rheumatoid arthritis, systemic lupus erythematosus, antiphospholipid syndrome, and Sjogren syndrome could be seen in a patient with ankylosing spondylitis.
